# Potential mechanisms and therapeutic targets of mesenchymal stem cell transplantation for ischemic stroke

**DOI:** 10.1186/s13287-022-02876-2

**Published:** 2022-05-12

**Authors:** Li Zhou, Huimin Zhu, Xue Bai, Jiagui Huang, Yue Chen, Jun Wen, Xuemei Li, Bowen Wu, Yongjun Tan, Mingfen Tian, Jiangxia Ren, Mengxia Li, Qin Yang

**Affiliations:** 1 Department of Neurology, The First Affiliated Hospital of Chongqing Medical University, 1 Youyi Road, Yuzhong District, Chongqing, 400016 China; 2 Department of Neurology, The First People’s Hospital of Neijiang, Sichuan, 64100 China

**Keywords:** Mesenchymal stem cells, Ischemic stroke, Stem cell transplantation, Therapeutic targets, Regenerative medicine

## Abstract

Ischemic stroke is one of the major causes of death and disability in the world. Currently, most patients cannot choose intravenous thrombolysis or intravascular mechanical thrombectomy because of narrow therapeutic windows and severe complications. Stem cell transplantation is an emerging treatment and has been studied in various central nervous system diseases. Animal and clinical studies showed that transplantation of mesenchymal stem cells (MSCs) could alleviate neurological deficits and bring hope for ischemic stroke treatment. This article reviewed biological characteristics, safety, feasibility and efficacy of MSCs therapy, potential therapeutic targets of MSCs, and production process of Good Manufacturing Practices-grade MSCs, to explore the potential therapeutic targets of MSCs in the process of production and use and provide new therapeutic directions for ischemic stroke.

## Introduction

Ischemic stroke is one of the major causes of death and disability in the world. Currently, thrombolysis and mechanical thrombectomy have revolutionized the treatment of ischemic stroke. However, there are serious imperfections, such as narrow time windows, risk of hemorrhage, issues of availability, and treatment failure [1, 2]. Therefore, it is very important to seek new alternatives with a wider time window and less hemorrhagic risk for ischemic stroke treatment to improve neurological function and reduce mortality. Stem cell‐based therapies are emerging as ideal candidates for functional recovery in stroke patients for the potential to reduce injury and enhance neurorestoration [3, 4].

At present, various types of stem cells, such as embryonic, induced pluripotent, neural, and mesenchymal stem cells (MSCs), as well as vascular and endothelial progenitor cells, have been used and investigated their curative potentials in the treatment of ischemic stroke [5]. Among them, bone marrow‐derived MSCs (BM‐MSCs) are the most commonly used MSCs for their easy‐to‐culture capabilities, safety, and weak immunogenicity. Moreover, growthing evidence indicates that MSCs affect the pathological processes of ischemic stroke via multiple targets and multitemporal, including reducing inflammation, modulating immune function, inhibiting apoptosis, promoting neurovascular, white matter, and synaptic remodeling in the acute, subacute, and chronic phases of ischemic stroke [4, 5]. Therefore, MSCs may be ideal “seed cells,” especially suitable for cell transplantation therapy of nervous system injury and degenerative diseases. However, many issues in the scientific refinement remain unresolved and require clarification. For example, choice of cell type, cell dose, Good Manufacturing Practices (GMPs)-grade production method at a reasonable cost for production, preservation, and transfer of the cells need to develop techniques that maximally enhance the effects of cell therapy on ischemic stroke [5, 6].

Here, we reviewed potential mechanisms and therapeutic targets of MSCs transplantation for ischemic stroke, including paracrine effector molecules, modulating immune function and remodeling of neurovascular unit, white matter and synapse, and the production process of GMPs-grade MSCs, looking forward to raising valid and general conclusions about the efficacy of MSCs in the treatment of ischemic stroke especially in clinical trials and providing new therapeutic directions for ischemic stroke.

## Overview of MSCs

MSCs were firstly identified by Friedenstein and his colleagues in 1970 and were named “mesenchymal stem cells” by Caplan in 1991. Subsequently, MSCs have many alternative names in the literature, including mesenchymal stromal cells, multipotent stromal cells, marrow stromal cells, mesodermal stem cells, and even medicinal signaling cells [7]. MSCs can be harvested from nearly any tissue type and have the potential of self-renewal and multidirectional differentiation, which can differentiate into mesenchymal tissues, including osteogenic, chondrogenic, adipogenic cells, and hematopoietic-supporting stromal cells [7, 8]. However, growthing animal and clinical studies showed that only a few transplanted MSCs in vivo were differentiated [8, 9]. Moreover, MSCs play a therapeutic role mainly through other pathways other than differentiation into tissue cells [8]. Thus, Caplan considered that MSCs are not “stem cells,” but rather Medicinal Signaling Cells as the therapeutic agents to illustrate their versatility and flexibility for adapting to the local microenvironment [8].

Currently, MSCs have become the most widely studied stem cell population and are widely used in clinical trials and/or treatment of various diseases, including blood diseases, liver, kidney and lung end-stage diseases, graft versus host disease, autoimmune diseases, various neurological diseases, and even the COVID-19 infected patients [10, 11].

## Safety, feasibility, and efficacy of MSC transplantation for ischemic stroke in animal models and clinical trials

### Studies in stroke animal models

During the acute phase of ischemic stroke in rats, MSCs therapy increased neuronal plasticity and functional recovery through protecting mitochondrial function, inhibiting apoptosis and pyroptosis of neurons, and reducing microglial activation in the peri-infarct area [12]. In the subacute phase of ischemic stroke in rats, GMP-grade human umbilical cord-derived MSCs (hUCMSCs) therapy effectively improved behavioral deficits, reduced infarct volume and glial scar formation, and promoted angiogenesis in ischemic penumbra [13]. In addition, in the subacute phase, hMSCs also reduced blood–brain barrier (BBB) disruption and apoptosis in the peri-infarct region via inhibiting pro-inflammatory cytokines and the M2-to-M1 macrophages/microglia phenotype shift [14]. In the chronic stroke rat model, transplantation of hUCMSCs maintained BBB integrity, attenuated behavioral deficits, and promoted neurogenesis and angiogenesis [15]. These studies suggest that MSCs therapy is safe, feasible, and effective for acute, subacute, and chronic ischemic stroke in rats.

### Studies in stroke clinical trials

In 2005, Bang et al. firstly, prospectively, and randomly examined the short-term follow-up feasibility, efficacy, and safety of culture-expanded autologous MSCs transplantation in 30 patients with cerebral infarcts within the middle cerebral arterial territory and severe neurological deficits. The patients in the MSCs group were infused intravenously 1 × 10^8^ autologous MSCs within 7 days of the stroke and followed for up to 1 year. The results showed that MSCs could promote functional recovery and were not reported any adverse events associated with transplantation [16]. In 2010, they also open-label, observer-blindly evaluated the long-term safety and efficacy of i.v. MSCs transplantation in 85 patients with severe middle cerebral artery territory infarct. Patients in the MSCs group were received i.v. autologous ex vivo cultured MSCs and followed for up to 5 years. The results also showed that MSCs were safe and could promote functional recovery depending on the specific characteristics of the patients, and associated with serum levels of stromal cell-derived factor-1 and the degree of involvement of the subventricular region of the lateral ventricle [17]. Subsequently, two phase II trials from Jaillard et al. and Law ZK et al. also confirmed the safety and feasibility of MSCs in subacute stroke [18, 19]. Similarly, studies for the treatment of chronic stroke have demonstrated the safety, feasibility, and therapeutic effects of MSCs [20]. These clinical trials also suggest that MSCs therapy is safe, feasible, and effective for acute, subacute, and chronic ischemic stroke in patients.

## Potential mechanisms and therapeutic targets of MSC transplantation for ischemic stroke

**Fig. 1:**
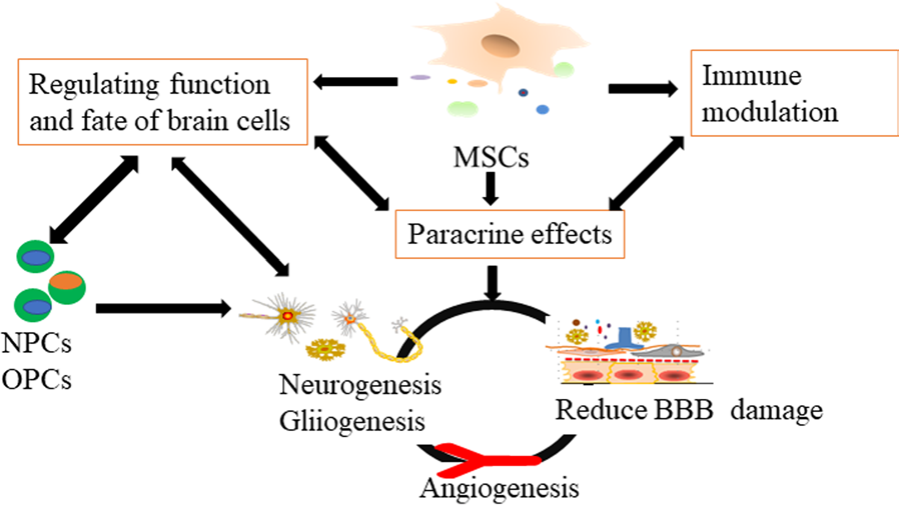
The possible therapeutic targets in MSCs transplantation therapy. *MSCs* mesenchymal stem cells, *NPCs* neural precursor cells, *OPCs* oligodendrocyte progenitor cells, *BBB* blood–brain barrier

Studies from animal models and clinical trials indicate that MSCs can effectively treat ischemic stroke. How do MSCs play a therapeutic role? What are the therapeutic targets for MSCs? As Fig. 1 shows, potential therapeutic targets of MSCs transplantation for ischemic stroke are listed.

### Paracrining effector molecules

MSCs can produce and secrete a large number of chemokines, cytokines, neurotrophic factors, and extracellular vesicles (EVs) by paracrine, commonly referred to as the MSCs secretome. These molecules and EVs would provide valuable insight on therapeutic targets of MSCs transplantation for ischemic stroke through multiple ways, such as anti-inflammation, anti-apoptosis, inhibition of fibrosis, promotion of angiogenesis and neurogenesis, immune regulation, and other functions [21]. We discuss these biological effector molecules in detail next, and they are further summarized in Table 1.

**Table 1:** Endocrine factors secreted by MSCs

Type	Secreted Factors	Function
Chemokines [23, 24]	CCL2-5, 7, 20, 26, CXCL1, 2, 5, 8, 10–12 and CX3CR1	Pro-angiogenesis; Immunoregulation; Improve the integrity of BBB; Promote the migration of different cells;
Cytokine [25–32]	IL-10	Anti-inflammation; Mediates microglia and macrophage phagocytosis
	IL-13	Anti-inflammation
	IL-3	Anti-inflammation; Pro-angiogenesis; Pro-neurogenesis; Promote cell survival, proliferation, and differentiation
	IL-1	Pro-inflammation; Pro-angiogenesis
	IL-8	Pro-inflammation; Pro-angiogenesis
	IL-6	Immunoregulation; Promote cell survival, proliferation, and differentiation
Growth and trophic factors [33–36]	BDNF	Pro-neurogenesis; Increase neuroplasticity; Promote cell survival (by inhibiting apoptosis)
	GDNF	Pro-angiogenesis; Pro-neurogenesis; Promote cell survival (by inhibiting apoptosis and autophagy); Improve the integrity of BBB
	VEGF	Pro-angiogenesis; Increase neuroplasticity; Pro-neurogenesis; Promote cell survival, proliferation, and differentiation
	TGF-β	Immunoregulation; Pro-neurogenesis; Involved in extracellular matrix remodeling; Promote cell survival, proliferation, and differentiation;
	bFGF	Pro-angiogenesis; Pro-neurogenesis; Promote cell survival, proliferation, and differentiation
	IGF-1	Anti-inflammation; Pro-neurogenesis; Improve the integrity of BBB; Promote cell survival, proliferation, and differentiation
	HGF	Anti-inflammation; Anti-apoptotic; Anti-fibrotic; Pro-angiogenesis; Pro-neurogenesis
	Ang-1	Pro-angiogenesis; Immunoregulation; Promote cell survival; Improve the integrity of BBB
MSC-Evs [37–39]	Express MSCs markers, such as CD29, CD73, CD90, CD44, CD105, and EV markers, such as CD107, CD63, CD9 and CD81. Pro-angiogenesis; Pro-neurogenesis; Promote e cell survival, proliferation, and differentiation; Immunoregulation; Increase neuroplasticity; Anti-fibrotic	

MSCs, mesenchymal stem cells; CCL, chemokine (C-C motif) ligand; CXCL, C-X-C ligand; CCR, CXCR, BBB, blood–brain barrier; IL, interleukin; BDNF, brain-derived neurotrophic factor; GDNF, glial cell line-derived neurotrophic factor; VEGF, vascular endothelial growth factor; bFGF/FGF-2, basic fibroblast growth factor; TGF-β, transforming growth factor-β; HGF, hepatocyte growth factor; and EVs, extracellular vesicles

#### Chemokines

The chemokine/chemokine receptor axis is indispensable for MSCs migration and immunoregulatory function, while MSCs can further promote the release of chemokines. Studies showed that cultured MSCs could release a variety of chemokines (such as C-C-motif ligand [CCL] 2–5, 7, 20, 26, C-X-C-motif ligand [CXCL] 1, 2, 5, 8, 10–12, and CX3CL1) and have chemokine receptors (such as C-C chemokine receptor [CCR] 1–5, 7, 9, C-X-C-motif receptor [CXCR] 3–6, and CX3CR1). These chemokines cooperate with MSCs to participate in the damage or repair process of ischemic brain tissue. For example, hypoxic preconditioning significantly increased the levels of CXCL8 and its receptor, CXCR2 in adipose tissue-derived MSCs (ADMSCs) dependent on mitogen-activated protein kinase (MAPK) activation in vitro, increased the homing ability of MSCs into the injured area [22]. Transplantation of MSCs overexpressing CCR2 could decrease ischemic lesions and improve the integrity of the BBB via a peroxiredoxin4-mediated antioxidant mechanism in vivo [23]. Taken together, MSCs have the ability to transmigrate and nest into the damaged/inflamed tissue via chemokines/chemokine receptor network, and strategies for enhancing infused MSCs homing to the ischemic brain might benefit the therapeutic promotion.

#### Cytokines

MSCs could release anti-inflammatory (such as interleukin-10 [IL-10], IL-13), pro-inflammatory (such as IL-8, IL-1α, IL-12), and pleiotropic cytokines (IL-6, IL-11, IL-16, IL-1β) to regulate immune function after ischemic stroke. In macrophages in vitro, UCMSCs increased the lipopolysaccharide-stimulated expression levels of IL-10 and IL-37 through PI3K/Akt signaling pathway to play an anti-inflammatory effect [24]. Further, transplantation of genetically engineered MSCs that overexpress IL‐10 (MSCs‐IL‐10) significantly increased autophagy, mitophagy, and cell survival markers, along with decreased markers for cell death and neuroinflammation than MSCs alone in vivo [25]. IL-8 had proangiogenic properties. Administration of IL-8-treated human bone marrow-derived MSCs (hBMSCs) increased angiogenesis in the infarct border zone and reduced infarction volume compared with the hBMSCs treatment alone in vivo [26]. Moreover, in a rat stroke model, IL-8 stimulated vascular endothelial growth factor (VEGF) production in hBMSCs in part via the PI3K/Akt and MAPK/ERK signal transduction pathways [27]. Treatment of pre-treated MSCs with IL-1α administered at the time of stroke reduced cerebral infarcted volume at 48 h and improved body mass gain, 28-point neurological score, and nest building [28]. Therefore, after administration of MSCs or cytokine-treated MSCs, significant changes in cytokines associated with the basic inflammatory pathology occur in the ischemic stroke brain and may serve as new therapeutic targets.

Toll-like receptor 9 (TLR9) agonist can stimulate MSCs to produce IL-6 *both *in vitro and in vivo. TSG-6-deficient MSCs displayed an increased capacity to release IL-6 conferring pro-inflammatory and pro-tumorigenic properties to the MSCs in mice [29]. On the contrary, the histone deacetylase inhibitor suberoylanilide hydroxamic acid decreased IL-6 secretion in IL-1β-induced MSCs through inhibition of the MAP3K4/NF-κB pathway in vitro [30]. IL‐11, a member of the IL‐6 cytokine family, enhanced proliferation and migration of ADMSCs, reduced apoptosis, and promoted cell survival by STAT3 signaling in vivo [31]. Thus, additional effort should be directed toward exploring and even balancing the bidirectional cytokine after ischemic stroke. New approaches based on modulation of bidirectional cytokine of MSCs hold promise for treatment of ischemic stroke.

#### Neurotrophic factors

In numerous ischemic stroke models, studies showed that expressions of neurotrophic factors, such as brain-derived neurotrophic factor (BDNF), glial cell line-derived neurotrophic factor (GDNF), VEGF, epidermal growth factor (EGF), and basic fibroblast growth factor (bFGF), were significantly increased in the MSC-treated animals compared with the non-treated animals in the infarcted borderline region. For example, transplantation of MSCs upregulated BDNF expression, reduced infarct area, increased neuronal survival, and improved functional outcomes [32]. Activated microglia could induce MSCs to produce GDNF and protect neurons against oxygen–glucose deprivation injury [33]. MSCs release of VEGF is mediated by both STAT3 and p38-MAPK following hypoxia or tumor necrosis factor (TNF) exposure [34]. Moreover, Wharton jelly-derived MSCs (WJMSCs) secrete a rich panel of trophic factors, such as bFGF, angiogenin, EGF, and sonic hedgehog, to promote angiogenesis [35]. Thus, treatment of MSCs will benefit the recovery of stroke, via increasing the release of these molecules to exert multiple roles (anti-inflammation, anti-apoptosis, inhibition of fibrosis, promotion of angiogenesis, and neurogenesis), which might be a potential target for the treatment of ischemic stroke.

#### MSC-extracellular vesicles

MSC-EVs can regulate injury and repair after ischemic stroke. Moreover, activated molecules in EVs are the key to neuroprotective effects. For example, MSC-EVs could promote post-stroke neuroregeneration and prevent postischemic immunosuppression in vivo [36], reduce microglial-mediated neuroinflammation, and enhance myelin maintenance after cortical injury in aged Rhesus monkeys [37]. Furthermore, MSC-EVs could release micro-RNA (miR)-133b, miR-184, miR-210, or miR-17–92 to promote neurogenesis, oligodendrogenesis, and angiogenesis, improve axonal or dendritic plasticity and neurite remodeling, regulate peripheral immunity, maintain microenvironmental homeostasis, and inhibit apoptosis in the cerebral ischemic rats [38]. Currently, many animal studies are attempting to evaluate the therapeutic potential of MSC-EVs for treatment of ischemic stroke. Future clinical trials should target the exosomes with consideration of safety and efficacy.

### Immunomodulatory effects of MSCs

**Fig. 2:**
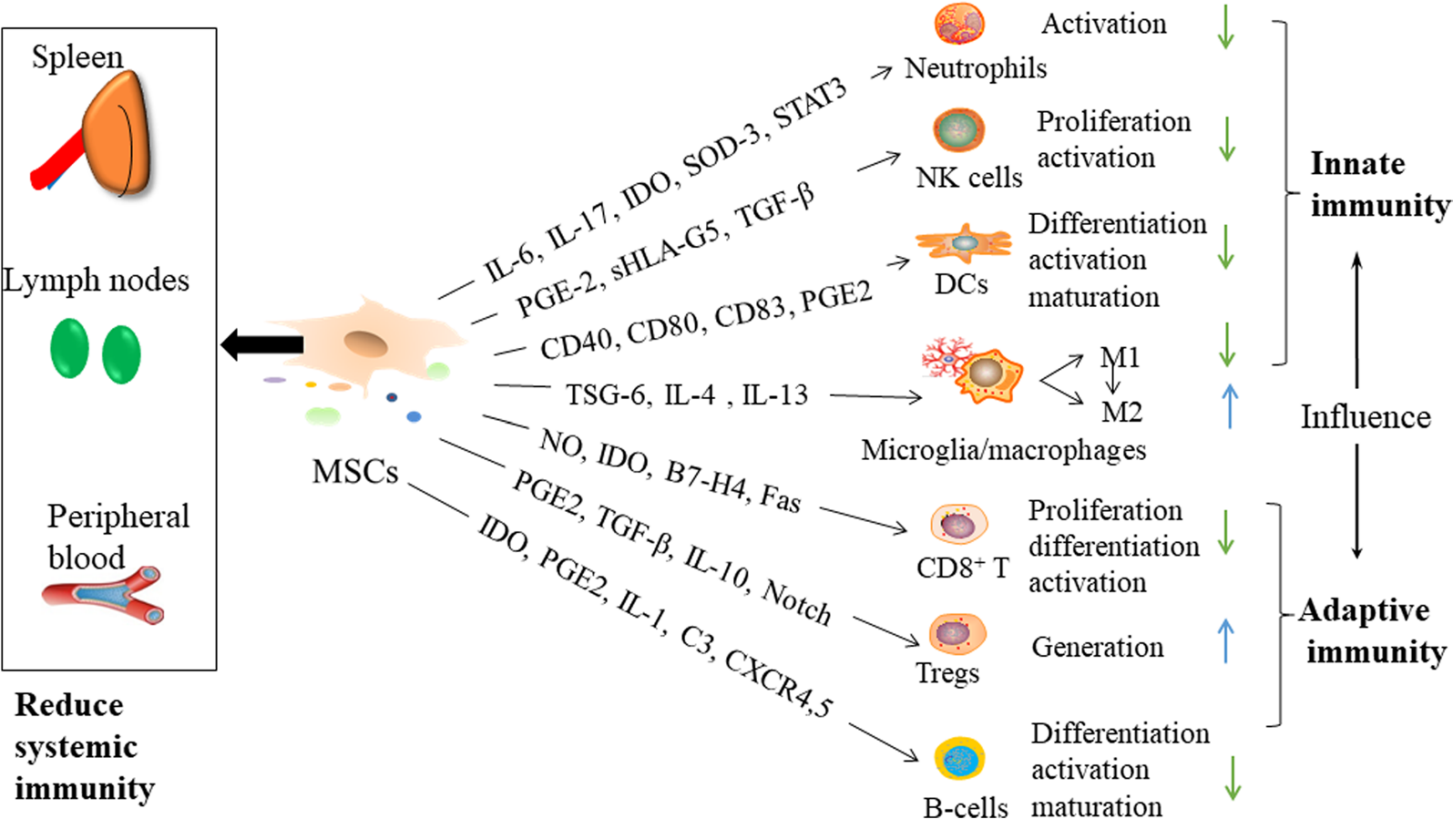
Potential targets of MSC-mediated immunomodulation. MSCs, mesenchymal stem cells; IL, interleukin; IDO, indoleamine 2,3-dioxygenase; SOD, superoxide dismutase; STAT, signal transducer and activator of transcription; PGE2, prostaglandin E2; sHLA-G5, soluble human leukocyte antigen-G5; TGF-β, transforming growth factor-β; CD, cluster of differentiation; TSG-6, tumor necrosis factor-α-stimulated gene-6; NO, nitric oxide; Fas, TNF receptor superfamily, member 6; C3, Complement 3; CXCR, C-X-C chemokine receptor; NK cells, natural killer cell; DCs, dendritic cells

Studies in vivo and vitro indicate that MSCs may regulate the immune by interacting with cells in the adaptive and innate immune systems to repair damaged brain tissue. The immunomodulatory function of MSCs is exerted through both cell–cell contact and the release of soluble factors. Figure 2 shows the immunomodulatory roles of MSCs.

#### Microglia/macrophage

Resident microglia in brain tissue can be activated firstly after ischemic stroke. Activated microglia are morphologically similar to macrophages which can be divided into two different subtypes, classically activated M1 and alternatively activated M2 type. M1-type microglia/macrophages release pro-inflammatory cytokines, pro-oxidant enzymes, and chemokines. In contrast, M2-type microglia/macrophages are involved in neuroprotection and secrete the anti-inflammatory molecules, which govern the resolution of post-ischemic inflammation and the release of growth factors implicated in brain repair.

MSCs have an important regulatory effect on microglia. By inhibiting TLR4 expression, MSCs can reduce activation of microglia. For activated microglia, MSCs can inhibit their proliferation and migration and reduce their phagocytosis [39]. Meanwhile, MSCs can promote apoptosis of microglia and protect the integrity of BBB. Moreover, MSCs treatment also decreased the number of activated microglia and expressions of pro-inflammatory cytokines and reactive oxygen species (ROS) [40], promoting microglia/macrophages to differentiate into M2-type both in vivo and vitro [40]. Therefore, MSCs can regulate the activation of microglia/macrophages to reduce neuroinflammation and brain tissue damage after ischemic stroke.

#### Innate immunity

Ischemic stroke destroyed the stability of BBB, which causes peripheral innate immune cells to infiltrate into brain tissue from blood circulation and further aggravates neuroinflammation. MSCs can regulate innate immune cells, including neutrophils, natural killer (NK) cells, dendritic cells (DCs), and macrophages.

Neutrophils are the first blood-borne immune cells through the damaged BBB to invade the ischemic tissue. MSCs transplantation can reduce the harmful effects of neutrophils in multiple ways. Firstly, MSCs may help to preserve the neutrophil reservoir in the bone marrow, significantly inhibit in vitro apoptosis of resting and IL-8-activated neutrophils by the IL-6 involved STAT3 pathway, and maintain effective functions and viability of neutrophils [41]. Secondly, MSCs prevent inappropriate and excessive activation of neutrophils and reduce the production of ROS in activated neutrophils through inhibiting ERK phosphorylation in vivo and vitro [41, 42]. Thirdly, MSCs can limit the intensity of a respiratory burst upon inflammatory stimulation and enhance the phagocytic activity of neutrophils by IL-17 secreting from MSC-stimulated memory T helper-17 (Th-17) cells in vitro, thereby helping to remove cell debris and eliminate infection and inflammation [43]. More importantly, MSC-small extracellular vesicles (sEVs) antagonize the detrimental effects of brain neutrophils without interfering with peripheral immune response after focal cerebral ischemia in rodents [44]. This indicates that the interaction between MSCs and neutrophils plays a critical role in reducing brain injury and neurologic deficits after ischemic stroke.

NK cells are a group of lymphocytes originating from the bone marrow. MSCs can inhibit proliferation and effective function of NK cells isolated from fresh peripheral blood. Moreover, hypoxic MSCs ameliorate limb ischemia in mice and have an increased ability of immunomodulation by reducing natural killer (NK) cytotoxicity and decreasing the accumulation of NK cells in vivo [45]. In vitro experiments indicate that MSCs can release prostaglandin E2 (PGE2), soluble human leukocyte antigen-G5 (sHLA-G5), and transforming growth factor-β (TGF-β) to inhibit the cytotoxicity of NK cells based on the upregulation of the NK cell activating receptor NKG2D [46]. Thus, MSCs can suppress the toxicity of NK cells and modulate the functions of NK cells that were associated with increased neovascularization and decreased inflammation and apoptosis at the peri-infract zone.

DCs can activate, maintain, and regulate the immune response. MSCs can inhibit differentiation and maturation of DCs as well as tilt mature DCs to immature states. On the one hand, after co-culture of MSCs and with DCs, MSCs can inhibit differentiation of CD14^+^ monocytes and CD34^+^ progenitor cells into DCs by inhibiting expressions of co-stimulating molecules, such as CD40, CD80, CD83, and CD86 [47, 48]. On the other hand, MSCs indirectly prevent differentiation of monocytes and stem cells into mature DCs by releasing PGE2 in animal model [42, 43]. Decreasing mature DCs reduce pro-inflammatory cytokines TNF-α and MHC II surface antigens [48] and increase anti-inflammatory cytokines IL-10 in vivo [47]. Hence, DCs are one of the targets of MSCs' immunosuppressive, which is worth further exploring in ischemia model.

#### Adaptive immunity

T cells are central to the adaptive immune system and harmful at the early stage after ischemic injury attributed to secretion of inflammatory cytokines and interaction with other cells. Pro-inflammatory T cells included T helper (Th 1), Th17, γδ T cells, and CD8+ T cells. In contrast to pro-inflammatory T cells, the immunosuppressive functions of anti-inflammatory T cells were impaired in ischemic stroke, such as regulatory T cells (Tregs) and Th2, which have neuroprotective effects on ischemic stroke [49].

In vitro experiments indicate that MSCs have direct immunosuppressive properties by inhibiting the activation and proliferation of CD4^+^ and CD8^+^ T cells via cell-to-cell contaction and elaboration of various soluble factors or B7-H4. Moreover, MSCs can suppress lysis of the target cells by mediation of CD8^+^ T cells [50]. MSCs also can inhibit effective function of naive and memory T cells and mediate apoptosis of previously activated T cells by upregulating Fas in vitro [51]. In addition, in critical limb ischemia patients, MSCs inhibit Th1 priming by diminishing expression of IL-12 [52]. In a translational ovine model of hypoxia-ischemia brain injury, researchers found that intravenously delivery MSCs reduced T-cell invasion, suppressed helper T-cell proliferation, and induced an anti-inflammatory, more tolerant, phenotype in these immune effector cells [53]. Thus, these findings suggest that immunoregulation of T cells by MSCs is highly plastic, and future studies should confirm the targets in ischemia model.

Tregs play a beneficial role in the pathogenesis of stroke. First, the onset of stroke directly decreases Tregs. Second, in the early stage of stroke, Tregs can elicit neuroprotective responses by enhancing clearance of immunological components and debris. Third, in the late stage of stroke, Tregs can suppress the proliferation and infiltration of effective T cells and the production of cytokines (TNFα, IL-6) [54]. MSCs can regulate activation, differentiation, proliferation, and other processes of Tregs through various mechanisms. For example, MSCs can induce differentiation of Tregs by enhancing PGE2, TGF-β, and IL-10 secretion in vivo [55]. For in vitro studies, TLR3 or TLR4 activation in MSCs promotes generation of Tregs via Notch pathway [56]. TGF-β produced by MSCs not only inhibits CD8^+^ T cells during infection expansion but also promotes development of Tregs, thereby suppressing T cell-dependent inflammation in rat renal ischemia/reperfusion injury [57]. Taken together, the combined effects of MSCs on inhibiting T cells proliferation and cytotoxicity and supporting production of Tregs can help control immune response.

B cells are the second main cell subset of the adaptive immune system. Activated and isotype-switched B cells infiltration worsens long-term outcomes after ischemic stroke. MSCs can inhibit activation, proliferation, differentiation, and chemotactic responses of B cells. For instance, MSCs can suppress B cells proliferation via inducing cell cycle arrest in G0/G1 phase and inhibit CXCR4- and CXCR5-mediated B-cell chemotaxis [58]. When MSCs were co-cultured with B cells extracted from peripheral blood, soluble factors secreted by MSCs inhibited B cells’ proliferation and production of immunoglobulin (IgM, IgG, and IgA) [59]. Beyond that, in the inflammatory environment, MSCs can indirectly regulate T cells and potential B cells by regulating innate immune cells, while B cell response is largely dependent on T cells [59]. Therefore, the interaction between MSCs and B cells in vivo may be significantly affected by MSCs-mediated inhibition to T cell function.

#### Systemic immunity

Increasing evidence indicated that stem cell therapy had not only neuroprotective effects on local microenvironment, but also modulated the splenic activation and peripheral immune responses. MSCs delivered by intracerebral/intravenous transplantation preferentially migrated to the spleen, reduced TNF-α expression in the spleen, and alleviated systemic inflammation [60]. In murine infection models, MSCs reduced bacterial levels in the alveoli, blood and spleen, increased anti-inflammatory cytokines levels, and reduced number of nucleated cells and neutrophils in serum [61]. Moreover, intravenous administration of MSCs inhibited lipopolysaccharide-induced acute lung and systemic inflammation and significantly reduced IL-6 levels and number of inflammatory cells [62]. Taken together, these findings suggest that transplantation of MSCs can modulate systemic immunity, decrease incidence of systemic infections, and improve the long-term sequelae of patient with ischemic stroke.

Above studies in vivo or vitro showed MSCs could regulate activation of microglia/macrophages, reduce excessive infiltration of neutrophils, balance functional status of T and B cell subsets in the inflammatory state, and decrease peripheral immune suppression by interaction with the peripheral immune system and incidence of systemic infections. Therefore, MSCs have immunomodulatory effects, which may help reduce damage and promote repair after ischemic stroke.

### Regulating function and fate of brain cells

**Fig. 3:**
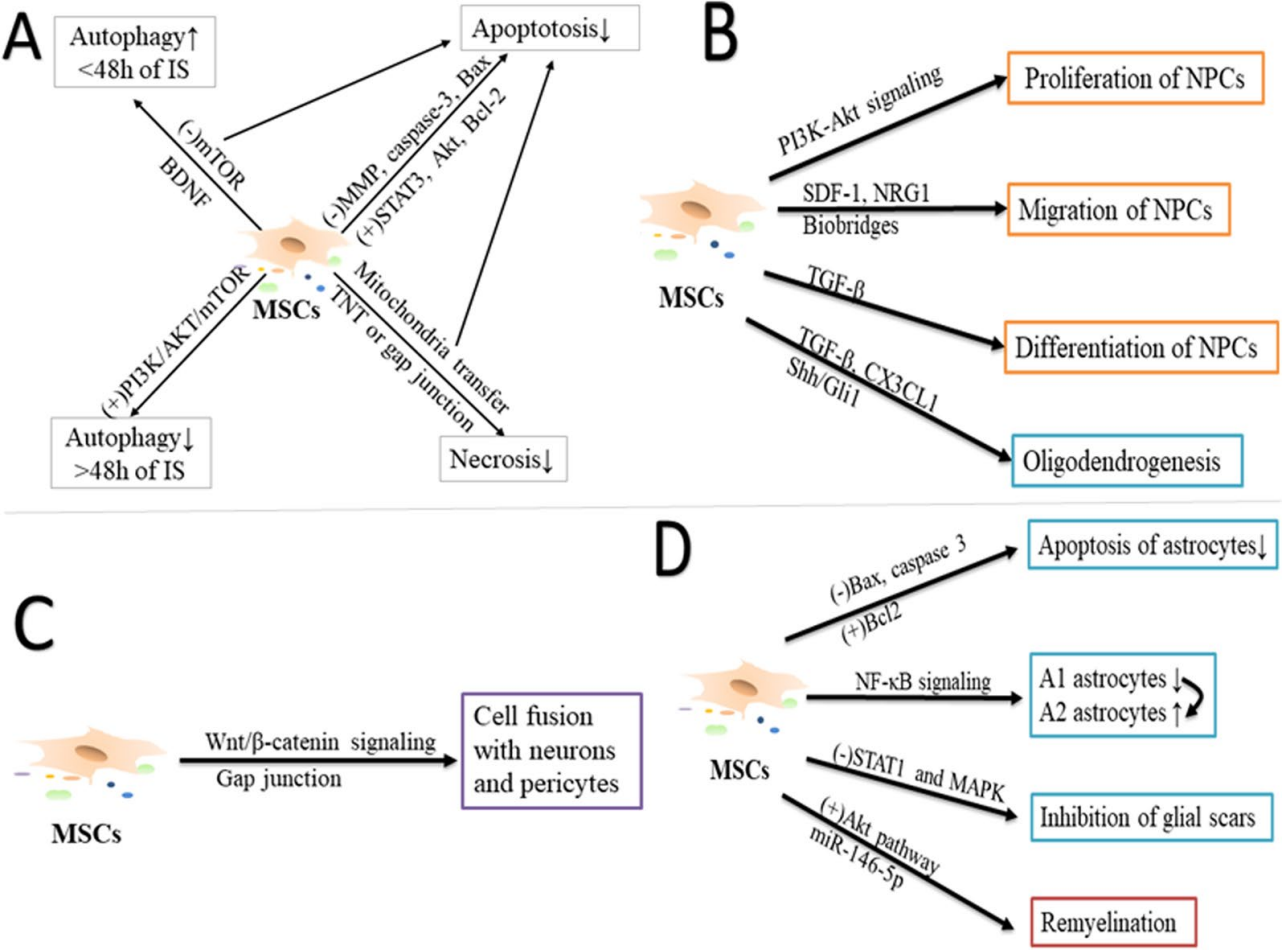
Potential targets of MSC-mediated regulating the function and fate of brain cells. **A** MSCs affect autophagy, apoptosis, and necrosis of brain cells to enhance cellular survival after ischemic stroke. **B** MSCs promote proliferation, migration, and differentiation of endogenous neural/oligodendrocyte precursor cells (NPCs/OPCs). **C** Cell fusion of MSCs with neurons and pericytes. **D** MSCs regulate function of glial cells. IS, ischemic stroke; MSCs, mesenchymal stem cells; mTOR, mammalian target of rapamycin; BDNF, brain-derived neurotrophic factor; MMP, matrix metalloprotease; STAT, signal transducer and activator of transcription; TNT, tunnel nanotube; PI3K/Akt, phosphoinositide-3-kinase/protein kinase B; SDF-1, stromal cell-derived factor-1; NRG-1, neuregulin 1; NPCs, Neural precursor cells; TGF-β, transforming growth factor-β; CX3CL1, C-X-3-C ligand-1; Shh/Gli1, sonic hedgehog/Gli1; NF-κB, nuclear factor kappa-light-chain-enhancer of activated B cells; and miR, micro-RNA

MSCs can also improve brain function by regulating the fate and function of brain cells [3]. In this section, we will describe the function of MSCs in regulating brain cell fate and function after stroke (Fig. 3).

#### Enhancing cellular survival

Autophagy has neuroprotective effect in the early stage of cerebral ischemia and becomes neurotoxic effect when cerebral ischemia lasts longer. MSCs transplantation can decrease cerebral ischemic injury by increasing autophagy at the early phase (within 24 h after ischemia) and enhance neuronal survival via inhibiting autophagy at the later period (48–72 h after ischemia). For example, by secreting BDNF and inhibiting the mTOR pathway activation, MSCs enhanced neuronal autophagy at 6, 12, and 24 h after oxygen–glucose deprivation in vitro, and at 12, 24, 48 h after hypoxic–ischemia in vivo, inhibited neuronal apoptosis, and attenuated cellular death and behavioral deficits [63]. At 72 h after cerebral ischemia, MSCs transplantation significantly inhibited neuronal autophagy, reduced cerebral infarct volume, and promoted behavioral recovery via activating the PI3K/Akt/mTOR signaling pathway [64].

Apoptosis is a form of cell death. MSCs transplantation can also inhibit apoptosis in many ways. For example, MSCs could inhibit apoptosis by regulating matrix metalloproteases and phosphorylation of STAT3 and Akt in vivo [64], inhibiting glutamate excitotoxicity and decreasing the levels of Ca^2+^ and surface GluR1 [65], or activating anti-apoptotic factor Bcl-2 and inhibiting endoplasmic reticulum stress and pro-apoptotic molecule Bax after cerebral ischemia [66].

MSCs can also rescue damaged cells through their mitochondria. Tseng et al. reported that MSCs mitochondria could be transferred to neurons at 24 h following hydrogen peroxide exposure in vitro, which was dependent on cell-to-cell contact. At the same time, neuronal viability was increased, neuronal metabolic function was significantly improved, and expression of mitochondrial outer membrane GTPase was upregulated [67]. Similarly, following cerebral ischemia in vivo, the host cells of injured cerebral microvasculature accepted the mitochondria transferred from transplanted MSCs, thereby significantly improving mitochondrial activity of injured microvasculature, enhancing angiogenesis, reducing infarct volume, and promoting functional recovery [68]. These studies indicated that MSCs could rescue damaged cells through transferring their own mitochondria into other cells.

#### Promoting proliferation, migration, and differentiation of endogenous neural/oligodendrocyte precursor cells

Cerebral ischemia can induce neurogenesis and oligodendrogenesis which is not enough to promote neurological recovery. MSCs can promote neurogenesis and oligodendrogenesis and improve neurological recovery. Shiota et al. reported that MSCs transplantation significantly increased endogenous neural precursor cells (NPCs) proliferation, migration, and differentiation in cerebral ischemic conditions by increasing chemokine and polysialylation enzyme expression [69]. MSCs also enhanced self-renewal and proliferation and differentiation of NPCs and suppressed inflammatory reaction by triggering the PI3K-Akt signal pathway [70]. Moreover, MSCs transplantation can increase adult hippocampal neurogenesis and improve schizophrenia-like behavioral phenotype [71]. In addition, interferon-γ (IFN-γ)-activated MSCs increased oligodendrogenesis and remyelination, inhibited microglial activation and microglial pro-inflammatory phenotype, and reduced infarct size by secreting nutritional factors, such as TGF-β and CX3CL1 [72].

#### Cell fusion

Cell fusion is the fusion of bone marrow-derived cells with local precursors or mature cells that transfer their genetic material and mix their cytoplasm. Studies indicate that MSCs can fuse with a variety of different cells, such as neurons, hepatocytes, cardiomyocytes, and even cancer cells. Moreover, MSCs can repair injured brain tissue through cell fusion. Kemp et al. reported that fusion of a bone marrow-derived cell with a neuron in vivo, in the mature brain, resulted in the formation of a spontaneously firing neuron. At the same time, fusion of MSCs with cerebellar Purkinje cells mitigated the effects of cell injury on electrical activity through Wnt/β-catenin signaling pathways [73]. Cell fusion may be involved in the formation of vascular tissue after stroke, and most of the fused cells in the penumbra can express pericellular markers (vimentin, desmin, CD45) [74]. Moreover, MSCs also have the potential to mediate cell–cell communication in gap junction, which initiates cell fusion. MSCs have an essential effect on the immunomodulatory and maintenance function of BBB through gap junction with endothelial cells [75]. Angiotensin II, insulin-like growth factor 1 pretreatment enhanced gap junction protein Cx43 expression and protected damaged cells and improved the therapeutic efficacy of MSCs transplantation during myocardial repair [76]. Therefore, MSCs can promote repair of brain tissue via fusing with neurons and pericytes after ischemic stroke.

#### Regulating function of glial cells

Glial cells are the most important subgroup of cells in brain tissue except neurons. Glial cells include astrocytes, microglia, and oligodendrocytes. Astrocytes are the most abundant glial cells in the central nervous system. A2 astrocytes exert protective effects by upregulating expressions of neurotrophic factors. A1 astrocytes are formed rapidly after the central nervous system injury and exert neurotoxic effects on myelin sheath, synapses, and neurons. MSCs transplantation exerts neuroprotective effects by preventing apoptosis of astrocytes and regulating the number of A2 astrocytes, inhibiting the formation of glial scars, preventing the inhibition of the axon regeneration via regulation of the NF-κB signaling pathway after ischemic stroke [77]. Moreover, MSCs reduced the release of pro-inflammatory cytokines by microglia as well as the activity of STAT1 and MAPK, which may consequently minimize astrogliosis and inhibit the formation of glial scars [77]. MSCs can not only regulate NPCs or oligodendrocyte progenitor cells (OPCs) differentiation, but also directly regulate function of oligodendrocytes. MSCs reduced death of oligodendrocytes in hypoxic-glucose conditions by producing growth factors and activating the Akt pathway in vitro. At the same time, MSCs promoted remyelination and protected axons from damage by producing and delivering miR-146-5p via exosomes [78]. These studies indicate MSCs can promote repair of ischemic cerebral tissue by regulating glial cells function.

## Production of good manufacturing practice-grade MSCs for ischemic stroke

Since Azizi et al. published the first report on the transplantation of human MSCs into rat brain in 1998, more and more studies on the treatment of neurological diseases by MSCs transplantation have been carried out. MSCs transplanted therapy is gradually shifting from laboratory to clinical therapy. Despite numerous registered clinical trials (http://www.clinicaltrial.gov/), there is no consensus on the manufacture of MSCs. The results obtained from clinical trials of ischemic stroke patients have been analyzed in meta-analysis studies [79]. Clinically, MSCs therapy in the brain is safe and improves primary clinical end points. However, MSCs, as advanced therapy medicinal product, require production and quality control in agreement with GMP (including management of facilities, staff training, manufacturing procedure, and quality control). It is often incomparable to compare the results from diverse clinical trials for the lack of validated, safe, and reproducible procedures in MSCs production and quality testing. Therefore, it is extremely difficult to raise valid and general conclusions about effects of MSCs therapy on ischemic stroke. Here, we will focus on the therapeutic targets-related manufacturing procedures and quality controls in GMP-grade MSCs production, to provide more possibilities for clinical transformation of MSCs in the treatment of ischemic stroke.

### Donor, cell sources, and culture processes

It is very important for the screening and testing of donor eligibility (mainly age and viral testing) during large-scale production of clinical-grade MSCs. The donor should be free from other abnormal risks that may be involved in MSCs. The age of the donor is also an important criterion because proliferation and multipotency of MSCs are directly associated with the age of the donor [80], and MSCs from children have a higher level of colony-forming unit-fibroblasts (CFU-Fs) [81].

Cell separation techniques represent an initial and very important step before stem cells are used in regenerative medicine, in vitro expansion and potential clinical applications. The main task for obtaining adequate material and increasing the therapeutic efficiency of MSCs [4, 82]. Currently, the most frequently reported sources of MSCs are bone marrow, adipose tissue, and neonatal and fetal tissue-derived MSCs (placenta, umbilical cord, and WJ). ADMSCs can express CD49d and produce more hepatocyte growth factor (HGF) and VEGF than BMSCs [83]. ADMSCs seem to display an increased proliferative potential and generate more rapidly a clinically effective cell dose compared with BMSCs in vitro [84]. But BMSCs are safer than ADMSCs, not only do not promote proliferation of existing tumors, but also release neurotrophic factors to promote repair of tissue. Recent research showed that clinical-grade MSCs from WJ complying with GMPs are simple, relatively fast and present a higher proliferation potential. Moreover, as availability of the source, WJ seems a recommended source for GMP production although their isolation can be difficult [85]. Therefore, it is critical to identify the most appropriate source of MSCs for the best desired effect, depending on the therapeutic application being considered.

The harvested cells were processed and centrifuged to obtain the stromal vascular portion containing MSCs. BM can be further enriched for MSCs with immunomagnetic devices or by fluorescence-activated cell sorting (such as stro1, CD49a, CD200, or CD271). In this process, plastic adherence remains the main step to isolate MSC populations. Subsequently, for clinical-scale production of MSCs, the use of a plating density of 1000 cells/cm^2^ is reasonable and allowed for a harvest of a high number of cells. Furthermore, for efficiency and safety reasons, limiting the number of population doublings to less than 20 should be reasonable [86].

The standard ex vivo expansion of MSCs is performed with either α-minimal essential medium or 10% fetal bovine (FBS), or 5% human serum or platelet lysates (hPL) from blood transfusion-secured sources. Currently, for availability and safety reasons, the most promising and most often used 5% PL, consisting of plasma enriched by platelet growth factors released by freezing–thawing cycles, represents an efficient alternative to FBS [87]. The effects of hPL are principally related to the presence of large quantities of cytokines and growth factors such as platelet-derived growth factor and FGF2, which supports MSCs’ ex vivo expansion without causing genomic instability. The immunophenotype, immunomodulatory potential, differentiation potential, and relative telomere length of MSCs remained unaffected by hPL [88].

### Controls for ex vivo-expanded MSCs

Cryopreservation maintains cell functional properties (e.g., immunomodulatory and differentiation ability) and survival rate of MSCs, allows pooling of cells to reach the cell numbers required for clinical application, and reduces biosafety risk [89]. However, before MSCs are transplanted into animals or patients, transient warming of freeze–thawed cells impairs expansion, viability, and potency of MSCs in vitro and disrupts partial cellular membrane which may alter signaling of IFN-γ receptors and down-regulate expression of indoleamine 2,3-dioxygenase (IDO) [90], impair immunomodulatory function of MSCs, enhance vulnerability to lysis by immune cells and complement system, and decrease in vivo persistence after intravenous administration [91]. A few days of rescue culture in vitro can eventually reduce this “cryo stun effect.”

Currently, a wide array of priming approaches on MSCs have been reported to improve their therapeutic efficacy, including hypoxia, 3D cultures, drugs, cytokines, and growth factors. For example, continuous low oxygen tension improved growth and genetic stability of MSCs by activating glycolysis [92]. 3D scaffolds could promote MSCs expansion and increase anti-inflammatory properties [40, 93]. Oligomycin or pro-inflammatory cytokines increased the immunosuppressive properties of MSCs by activating AMPK signaling [94]. Metabolic reprogramming of GMP-grade hUCMSCs increased their suppressive potential in acute graft vs. host disease [95].

Although there were many means for upscaling MSCs culture and bringing MSCs to clinical application, it has yet to be standardized for economical and feasible approaches that meet GMP compliance.

### Potency assays of MSCs

In advanced clinical trials, potency assays of MSCs are essential, which included certain surface markers, differentiative potential, senescence status, secretome and immunomodulatory functions of MSCs [4]. Phinney et al. established the “Clinical Indications Prediction Scale” (CLIP) and reported that transcription factor Twist1 regulated functions of MSCs. In other words, MSCs with high expression of Twist1 are characterized by rapid growth, high CFU-Fs activity, a low intrinsic level of apoptosis, and a pro-angiogenic phenotype, whereas MSCs with low expression of Twist1 are characterized by anti-inflammatory and immunomodulatory activities [96]. Chinnadurai et al. estimated the immunomodulatory capacity between MSCs and peripheral blood mononuclear cells in vitro with quantitative RNA-based array and secretome analysis. The results showed that MSCs suppressed T cell proliferation via large mediation of IDO, which was correlated with the secretion and expression of CXCL9, CXCL10, VEGF, and CCL2 [97]. In MSCs-peripheral blood mononuclear cell co-culture settings, the loop analytical method showed that phosphorylation of STAT1 and STAT3 in MSCs was correlated with T-cell suppression [98]. Therefore, the potency assays for verifying MSCs identity and quality should be more explored by combining MSCs heterogeneity, molecular markers, and the high complexity of the local microenvironment. It also needs to further elucidate combinatorial potency assays based on the increasing knowledge of MSCs biology.

Here, we expound on the production of GMP-grade MSCs for ischemic stroke. Results of existing studies provide data on the safety and feasibility of stem cell therapy. Due to differences in patient population, cell origin, time of administration, and drug delivery systems, it is needed to solve the challenging point of definition and implementation of relevant controls for safety, particularly for testing genetic stability and rigorous potency assays, to standardize MSCs therapy and avoid outcome bias.

## Conclusions

**Fig. 4:**
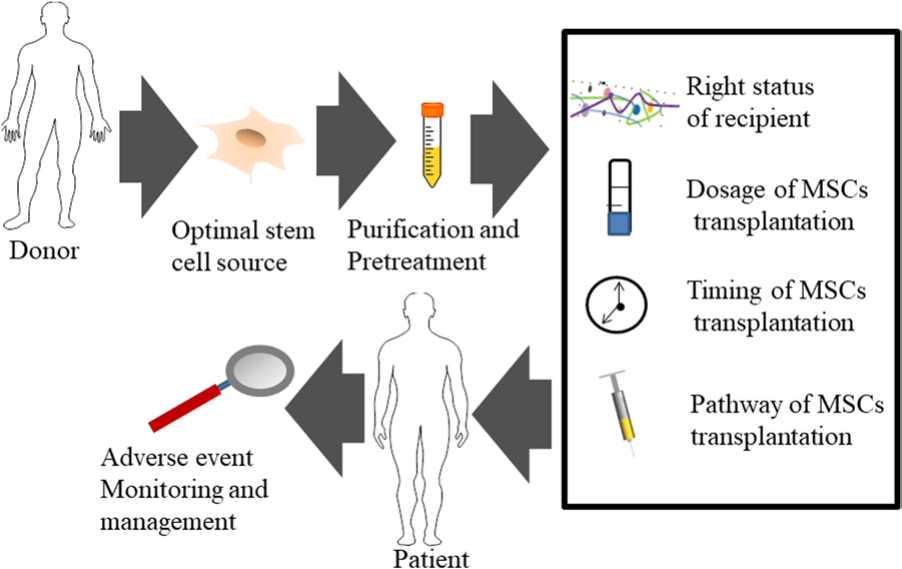
Key considerations for MSC-based clinical applications. *MSCs* mesenchymal stem cells

The potential of MSCs in the treatment of ischemic stroke is huge. In this article, we reviewed the potential therapeutic targets of MSCs in ischemic stroke therapy and also discussed how logistical and other requirements of GMP-based MSCs production. A large body of preclinical and clinical work supported the safety and restorative effects of MSCs transplantation. However, many key issues, such as optimal cell source, preparation of MSCs in full compliance with GMPs, dosage, transplantation time window and pathway, and adverse event monitoring and management, must be resolved before clinical application because each ischemic stroke patient has different physiological conditions (Fig. 4). Therefore, to solve the above problems, it is urgent to elucidate the mechanism of MSCs therapy for ischemic stroke. In addition, it is also necessary to explore paracrine effects, interaction between various soluble cytokines, and modulation of multicellular fate in MSCs, which may be a key part of MSCs' therapeutic potential. Moreover, the production and delivery of MSCs should conform to European GMPs (Euralex) to facilitate multicenter trials.

In conclusion, it seems that MSCs can be utilized as a therapeutic candidate in stroke therapy and pave the way for new treatments in the near future, to improve neurologic function, survival, and quality of life for ischemic patients.

## Data Availability

Please contact the corresponding author for data requests.

## Abbreviations

MSCs: Mesenchymal stem cells
GMPs: Good Manufacturing Practices
tPA: Tissue plasminogen activator
hUCMSCs: Human umbilical cord-derived MSCs
BBB: Blood–brain barrier
EVs: Extracellular vesicles
CCL: Chemokine (C-C motif) ligand
CXCL: C-X-C ligand
CCR: C-C chemokine receptor type
CXCR: C-X-C chemokine receptor
MAPK: Mitogen-activated protein kinase
IL: Interleukin
hBMSCs: Human bone marrow stromal cells
VEGF: Vascular endothelial growth factor
PI3K/AKT/mTOR: Phosphoinositide-3-kinase/protein kinase B/mammalian target of rapamycin
MAPK/ERK: Mitogen-activated protein kinase/extracellular regulated protein kinases
TLR: Toll-like receptor
TSG-6: Tumor necrosis factor-α-stimulated gene-6
NF-κB: Nuclear factor kappa-light-chain-enhancer of activated B cells
STAT: Signal transducer and activator of transcription
ADMSCs: Adipose tissue-derived MSCs
BDNF: Brain-derived neurotrophic factor
GDNF: Glial cell line-derived neurotrophic factor
EGF: Epidermal growth factor
bFGF/FGF-2: Basic fibroblast growth factor
TNF: Tumor necrosis factor
WJMSCs: Wharton jelly-derived MSCs
miRNA: Micro-RNA
ROS: Reactive oxygen species
NK cell: Natural killer cell
DCs: Dendritic cells
Th-17 cells: T helper-17 cells
PGE2: Prostaglandin E2
sHLA-G5: Soluble human leukocyte antigen-G5
TGF-β: Transforming growth factor-β
TNF-α: Tumor necrosis factor-α
Tregs: Regulatory T cells
IFN-γ: Interferon-γ
NPCs: Neural precursor cells
OPCs: Oligodendrocyte progenitor cells
CFU-Fs: Colony-forming unit-fibroblasts
HGF: Hepatocyte growth factor
FBS: Fetal bovine
hPL: Human platelet lysates
IDO: Indoleamine 2,3-dioxygenase
CLIP Scale: Clinical Indications Prediction Scale
